# A Novel Polysaccharide Conjugate from *Bullacta exarata* Induces G1-Phase Arrest and Apoptosis in Human Hepatocellular Carcinoma HepG2 Cells

**DOI:** 10.3390/molecules22030384

**Published:** 2017-03-01

**Authors:** Ningbo Liao, Liang Sun, Jiang Chen, Jianjun Zhong, Yanjun Zhang, Ronghua Zhang

**Affiliations:** 1 Department of Nutrition and Food Safety, Zhejiang Provincial Center for Disease Control and Prevention, Hangzhou 310051, China; nbliao@cdc.zj.cn (N.L.); lsun@cdc.zj.cn (L.S.); yjzhang@cdc.zj.cn (Y.Z.); 2 College of Biosystem Engineering and Food Science, Zhejiang University, Hangzhou 310058, China; bedelight@163.com

**Keywords:** *Bullacta exarata*, polysaccharide, human hepatocellular carcinoma HepG2 cells, apoptosis

## Abstract

*Bullacta exarata* has been consumed in Asia, not only as a part of the normal diet, but also as a traditional Chinese medicine with liver- and kidney-benefitting functions. Several scientific investigations involving extraction of biomolecules from this mollusk and pharmacological studies on their biological activities have been carried out. However, little is known regarding the antitumor properties of polysaccharides from *B. exarata*, hence the polysaccharides from *B. exarata* have been investigated here. One polysaccharide conjugate BEPS-IA was isolated and purified from *B. exarata*. It mainly consisted of mannose and glucose in a molar ratio of 1:2, with an average molecular weight of 127 kDa. Thirteen general amino acids were identified to be components of the protein-bound polysaccharide. Methylation and NMR studies revealed that BEPS-IA is a heteropolysaccharide consisting of 1,4-linked-α-d-Glc, 1,6-linked-α-d-Man, 1,3,6-linked-α-d-Man, and 1-linked-α-d-Man residue, in a molar ratio of 6:1:1:1. In order to test the antitumor activity of BEPS-IA, we investigated its effect against the growth of human hepatocellular carcinoma cells HepG2 in vitro. The result showed that BEPS-IA dose-dependently exhibited an effective HepG2 cells growth inhibition with an IC_50_ of 112.4 μg/mL. Flow cytometry analysis showed that BEPS-IA increased the populations of both apoptotic sub-G1 and G1 phase. The result obtained from TUNEL assay corroborated apoptosis which was shown in flow cytometry. Western blot analysis suggested that BEPS-IA induced apoptosis and growth inhibition were associated with up-regulation of p53, p21 and Bax, down-regulation of Bcl-2. These findings suggest that BEPS-IA may serve as a potential novel dietary agent for hepatocellular carcinoma.

## 1. Introduction

Since ancient times, *Bullacta exarata*, a kind of shell mollusk native of Asia, has been consumed by humans, not only as a part of the normal diet, but also as a delicacy because they have a highly desirable taste and aroma. In addition, the nutritional, tonic, and medicinal properties of *B. exarata* have been recognized for a long time [1]. We previously reported that a *B. exarata* polysaccharide (BEPS-IB) showed much more effective antioxidant activity in scavenging superoxide radicals in vitro [2]. Furthermore, Zhang et al. showed that BEP-3, a bioactive polysaccharide, exhibited antitumor activity against human pancreatic cancer SW1990 cells, human breast cancer Bcap37 cells and human HeLa cervical cancer cells [3]. Recently, some studies have also shown that the extracts of *B. exarata* possess a broad range of biological activities including hepato-protective, antioxidant, anticancer, antihypertension, and hypocholesterolemic effects [2,3,4,5,6]. However, the active constituents of *B. exarata* have not been studied extensively.

Hepatocellular carcinoma (HCC) characteristically presents with a poor prognosis and is one of the major leading causes of cancer death worldwide, especially in Eastern Asia [7]. At present, HCC is the eighth most common malignancy in women, the fifth most common malignancy in men worldwide and the number of incident cases and deaths continues to increase year by year [8]. Although knowledge about HCC is expanding exponentially in recent years, treatment and prevention of HCC are still a big challenge. On diagnosis most HCC patients are terminal. Despite the fact that chemotherapy is a common therapeutic strategy after surgery, its application is hindered by growing multidrug resistance in tumor cells and limited due to its serious adverse effects. Therefore, finding new nontoxic chemo-preventive or chemotherapeutic agents with fewer side effects is essential.

Polysaccharide-protein complexes are biopolymers comprised of the protein or polypeptide covalently linked together with carbohydrates as the side-chains. These structures can be linear or contain branched side chains. Polysaccharide-protein complexes were obtained from different sources, such as algae, plants, microorganisms, and animals. Previous studies had revealed that polysaccharide-protein complexes can inhibit the tumor cells growth in vitro by activating multiple signal pathways including cell cycle arrest, DNA damage, and alteration of death inhibitors or promoters expression [9]. In vitro experiments, *Pleurotus abalonus* polysaccharide-peptide LB-1b, *Streptococcus pyogenes* glycoprotein SAGP, *Hypsizigus marmoreus* glycoprotein HM3A and *Lycium barbarum* polysaccharide-protein complex could kill the tumor cell lines (liver, lymphoma, leukemia, stomach, and lung) directly via inducing cell cycle arrest, up-regulating Bax gene expression and/or down-regulating Bcl-2/Bcl-xl genes expression [10,11,12,13,14]. However, the antitumor effects of polysaccharide-protein complexes upon liver cancer have not yet been fully investigated. Moreover, the mechanism of direct inhibition of cell proliferation has also not been completely elucidated.

In the present study, a novel polysaccharide–protein complex (BEPS-IA) was extracted and purified from *B. exarata*. Then the chemical structures were analyzed. Finally, we have investigated the antitumor activity of BEPS-IA against human hepatocellular carcinoma HepG2 cells, and its role on cell cycle arrest and apoptosis of HepG2 cells, and reveal a possible signal pathway for elucidating the BEPS-IA′ anticancer molecular mechanisms.

## 2. Results and Discussion

### 2.1. Composition Analyses and Image of BEPS-IA

**Figure 1:**
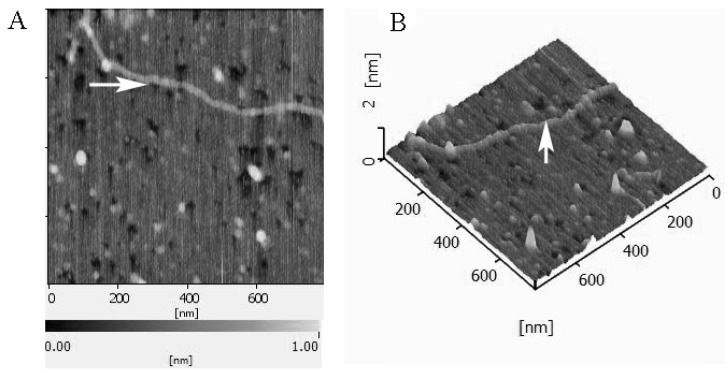
Atomic force microscopy (AFM) image of *B. exarata* polysaccharide-protein complex BEPS-IA was obtained with a Nano Scope III atomic force microscope and collected by tapping mode atomic force microscopy. The BEPS-IA concentration was 1 μg/mL. (**A**) Topographic image of a 90 min BEPS-IA sample taken by AFM in air at room temperature; (**B**) 3D visualization of BEPS-IA polymer reveal thin and thick polymer portions corresponding to areas highlighted with arrows in (**A**).

The chemical compositions and contents of carbohydrate, protein, M_W_ and sulfonic acid in BEPS-IA have been reported previously (Table S1) [2]. In this study, the AFM image of BEPS-IA is shown in Figure 1. The fiber-like structure and small branches of BEPS-IA molecule can be observed. The chain length of the backbone was approximately 948–1032 nm and the height was 53–67 nm. Monosaccharide composition analysis, carried out by HPLC following acid hydrolysis and derivatization with PMP, showed that BEPS-IA was composed of primarily of glucose and mannose, with a molar ratio of about 2:1, whereas others (e.g., galactose (Gal), and arabinose) were in a minor content. The amino acid composition in the polysaccharide-protein complex was analyzed by the HPLC AccQ method. As shown in Table 1, thirteen amino acids were identified to be components of the macromolecule. BEPS-IA was rich in glycine (22.14 mg/g), followed by asparagine (16.73 mg/g), glutamic acid (12.27 mg/g), alanine (9.26 mg/g) and valine (8.95 mg/g).

### 2.2. UV and IR Spectrum of BEPS-IA

**Figure 2:**
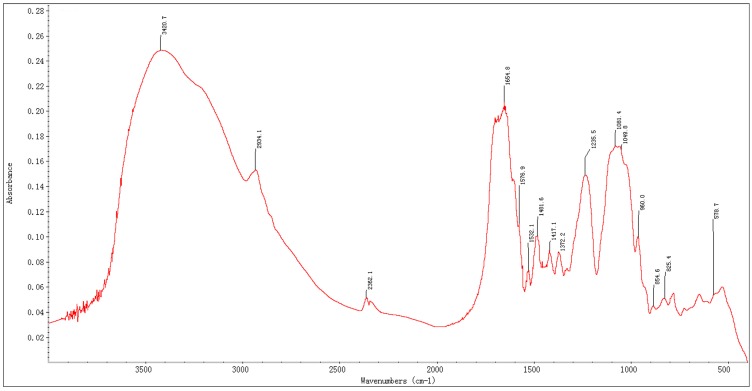
FT-IR spectrum of *B. exarata* polysaccharide-protein complex BEPS-IA.

The absorption peak at 280 nm in the UV spectrum, indicated that BEPS-IA contained protein portions. The IR spectrum of BEPS-I exhibited some characteristic absorption peaks of polysaccharide. As shown in Figure 2, the peak at 3420.7 cm^−1^ would be due to the stretching vibration of O–H and/or N–H. The peak at 2934.1 cm^−1^ would be due to the stretching vibration of C–H. The band at 1654.8 cm^−1^ was attributed to the stretching vibration of C–O and/or the variable-angle vibration of N–H. The band at 1481.6 cm^−1^–1372.2 cm^−1^ was attributed to the stretching vibration of C–O, and the band at 1341.1 cm^−1^–1235.5 cm^−1^ was attributed to the stretching vibration of O–H [15]. The characteristic absorption at 876.1–904.8 cm^−1^ would be due to the presence of β-glycosidic bonds in BEPS-IA [16]. Moreover, absorption bands at 820.2, and 842.4 cm^−1^ indicated the presence of mannose and glucose in BEPS-IA. A characteristic absorption at 854.6 cm^−1^ was also observed, indicating the α-configuration of BEPS-IA units [16]. All these findings indicated the possibility that the polysaccharide BEPS-IA mainly composed of mannose and glucose, containing both α-and β-glycosidic bonds, is a proteoheteroglycan.

### 2.3. Structure Data from NMR

**Figure 3:**
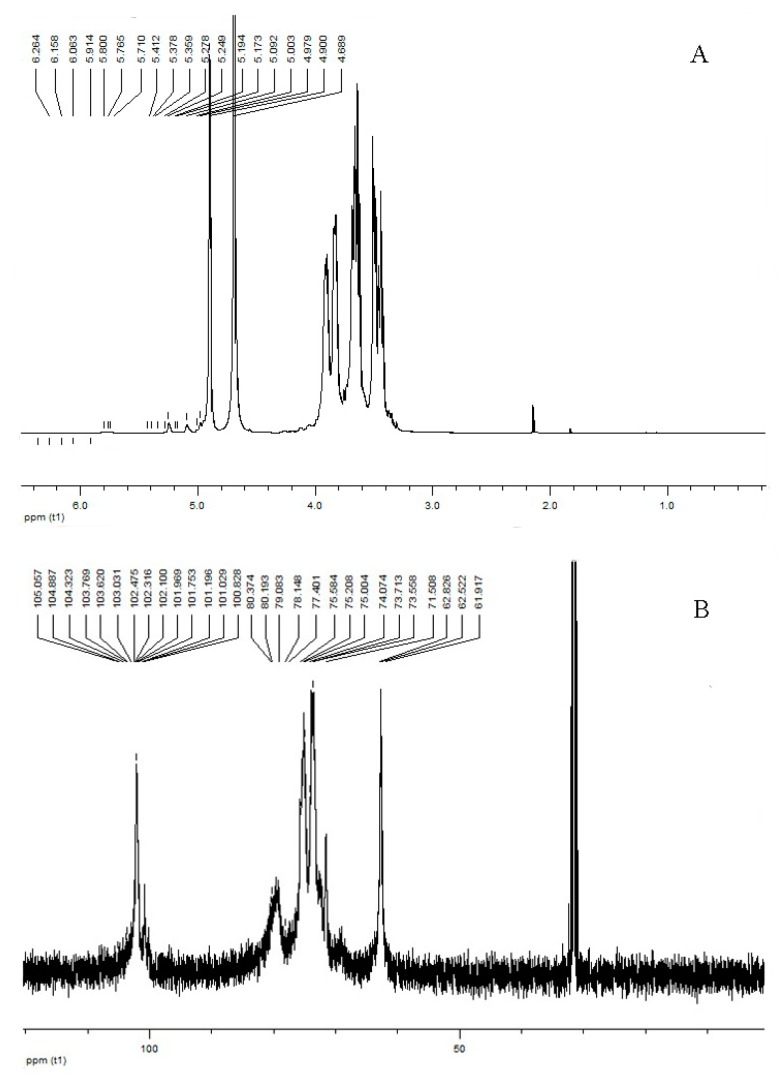
NMR spectra of the *B. exarata* polysaccharide-protein complex BEPS-IA in D_2_O. (**A**) ^1^H-NMR and (**B**) ^13^C-NMR.

NMR spectroscopy has become the most powerful technique for the structure analysis of carbohydrates. As shown in Figure 3A, the ^1^H spectrum showed peaks in the anomeric region at 4–6 ppm. In this spectrum, signals 4.82, 4.91, 5.01 and 5.17 ppm, implied that the sugar rings of BEPS-IA were pyranose rings and the sugar residues of BEPS-IA were connected by α- and β-configuration glycosidic bonds [16].

There were also signals of other sugar protons at 3.30–4.31 ppm. According to Dore et al., 2014 [17], the chemical shifts correspond to C2 and C6. We also observed that the regions between 1.6 and 2.4 are related to the glucan-protein structure. These observations are in accord with other studies [17]. In addition, the ^13^C-NMR spectrum of BEPS-IA (Figure 3B) had no signal at low field from 160 to 180 ppm, which illustrated that it did not contain uronic acid. Based on the available data in the literature [18], the resonances of 99.28–105.06 ppm were attributed to the anomeric carbon atoms of mannopyranose (Manp) and glucopyranose (Glcp), respectively. Other important signals in the spectra are those in the 60–80 ppm range, where they are related to C2 (73.71 ppm), C3 (84.3 ppm), C4 (80.0 ppm), C5 (76.18 ppm) and C6 (61.92 ppm) of that carbohydrate [15,16]. The 47.8 ppm signal is related to the –CH_2_N group of amino acids. The presence of additional peaks in the range of 20–40 ppm may suggest the presence of a glucan–protein structure [18]. These NMR results were consistent with those of the FTIR spectrum.

### 2.4. Structure Data from Methylation Analysis

**Figure 4:**
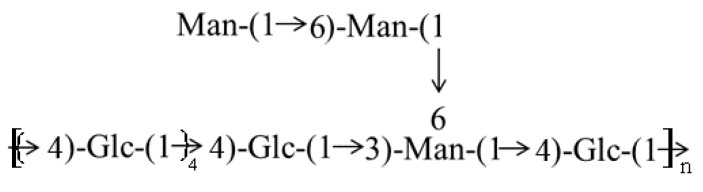
Proposed structural feature of the BEPS-IA isolated from *B. exarata*.

The fully methylated BEPS-IA was hydrolyzed with acid, converted into alditol acetates, and analyzed by GC–MS. As shown in Table 2, the presence of four major compounds, 2,3,6-Me_3_-Glc, 2,4-Me_2_-Man, 2,3,4-Me_3_-Man and 2,3,4,6-Me_4_-Man, which indicate the presence of 4-linked Glc, 3,6-linked Man, 6-linked Man, and terminal Man, in a molar ratio of about 6:1:1:1. The high proportion of 4-linked Glc indicates that the main consecutive repeating unit of BEPS-I is (1→4)-linked Glc. On the basis of above-mentioned results, the monomer of BEPS-IA repeat unit was determined to be as illustrated in Figure 4 below.

### 2.5. Anti-Proliferation and Cytotoxicity Assay

**Figure 5:**
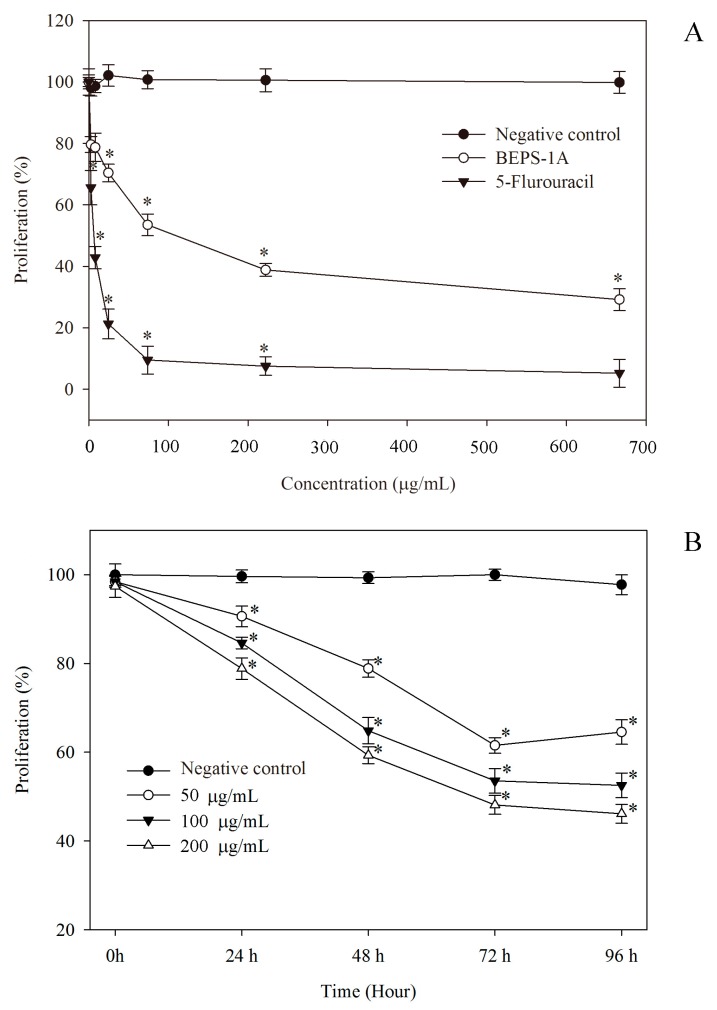
Growth inhibitory effect of B. exarata polysaccharide-protein complex BEPS-IA on HepG2 cells. (**A**) HepG2 cells were treated with various concentrations of BEPS-IA for 72 h; (**B**) The antiproliferative activity of BEPS-IA (0, 50, 100 and 200 μg/mL, respectively) on the HepG2 cells growth at 24, 48, 72 and 96 h. Untreated cells (0 μg/mL) group was used as negative control and 5-fluorouracil (5-FU) group was used as a positive control. The data were expressed as mean ± SD of three experiments. Values marked with * are significantly different compared to the control (*p* < 0.05).

In this study, the anti-proliferation activity of BEPS-IA was tested on human hepatocellular carcinoma HepG2 Cells, and the common antitumor drug 5-FU was used as a positive control. Figure 5A shows that BEPS-IA and 5-FU displayed dose dependent inhibition effects on HepG2 cells. The amount of BEPS-IA and 5-FU required to inhibit the proliferation of HepG2 cells by 50% (IC_50_) was 112.4 ± 22.53 and 23.94 ± 5.17 μg/mL, respectively. BEPS-IA (from 0 to 200 μg/mL) dose- and time-dependently inhibited the proliferation of HepG2 cells (Figure 5). With 200 μg/mL of BEPS-IA, the proliferation of the HepG2 cells was inhibited by 56% after 72 h. To further evaluate the antiproliferative effects of BEPS-IA on human hepatocellular carcinoma HepG2 cells, the dose of BEPS-IA was increased from 200 to 700 μg/mL. At concentrations >200 μg/mL, BEPS-IA also dose-dependently inhibited HepG2 cell proliferation. At the highest dose (700 μg/mL), HepG2 cell proliferation was inhibited by about 74.3% (Figure 5A). However, the study of cytotoxicity against HepG2 cells by the BEPS-IA showed that the polysaccharide, above a dose of 200 μg/mL, exhibit cytotoxicity toward HepG2 cells in vitro (Cytotoxicity was determined by a 10% reduction of absorbance at 570 nm reading for each concentration compared to the control), as shown in Figure S1. Therefore, the low doses of BEPS-IA (from 0 to 200 μg/mL) were chosen for the subsequent experiments.

### 2.6. Effect of BEPS-IA on Cell Cycle Distribution of HepG2 Cells

**Figure 6:**
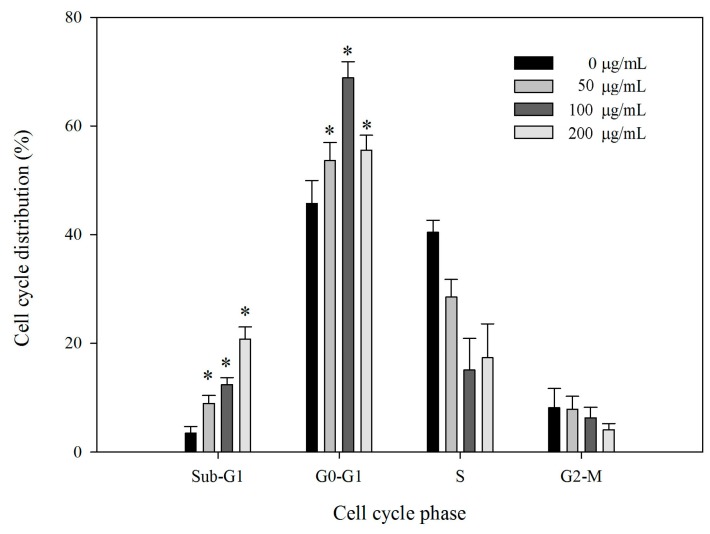
BEPS-IA induced cell cycle arrest in HepG2 cells. Cells were cultured for 24 h with BEPS-IA (50, 100 and 200 μg/mL, respectively). Bar graph summarizes the data on cell cycle distribution from three independent experiments. Values marked with * are significantly different compared to the control (*p* < 0.05).

To estimate the effect of BEPS-IA treatment on the distribution of cells in the cell cycle, we performed DNA cell cycle analysis by flow cytometry (Figure S2). The sub-G1 cell fraction was considered to represent apoptotic cells. As shown in Figure 6, the results revealed that BEPS-IA produced 2.1-, 2.8-, and 6.5-fold increases in the apoptosis of HepG2 cells at concentrations of 50, 100, and 200 μg/mL, respectively, compared with that induced with 0 μg/mL BEPS-IA (Control). The effects of BEPS-IA treatment for 24 h on the HepG2 cell-cycle phase distribution are also observed. Compared with the control (41.2%), treatment with 50, 100, or 200 μg/mL BEPS-IA for 24 h increased the population of cells in G1 phase to 62.3%, 74.2%, or 46.8%, respectively. These results showed that BEPS-IA treatment of HepG2 cells induced the accumulation of G1 phase cells. We also found that as the concentration of BEPS-IA increased, the sub-G1 apoptotic fraction of cells increased significantly from 3.2% to 22.8%. Therefore, the induction of G1-phase arrest and an increased sub-G1 apoptotic fraction may be the major mechanisms by which the growth of HepG2 cell is inhibited.

### 2.7. BEPS-IA Induced Apoptosis of HepG2 Cells In Vitro

**Figure 7:**
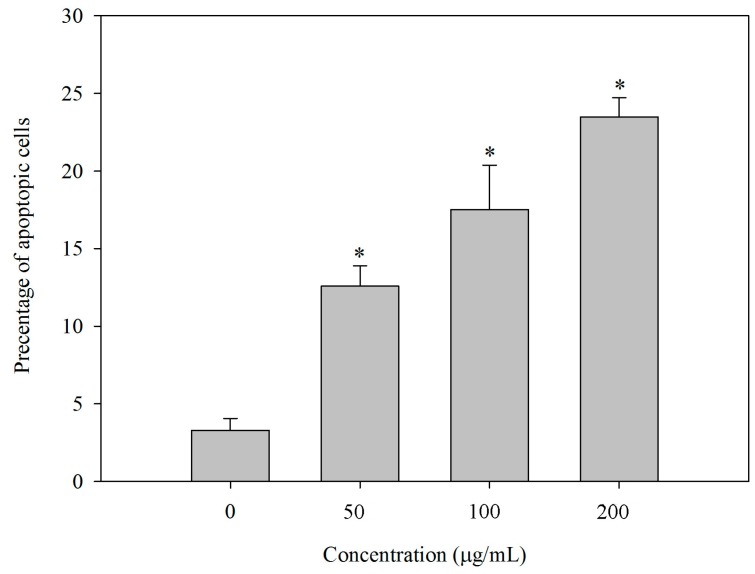
Apoptotic rate (%) of human hepatocellular carcinoma HepG2 cells treated with polysaccharide BEPS-IA. Cells were cultured for 24 h with BEPS-IA (50, 100 and 200 g/mL, respectively). Untreated cells (0 g/mL) were used as control. Values marked with * are significantly different compared to the control (*p* < 0.05).

To further evaluate the effect of BEPS-IA on the induction of apoptosis, the HepG2 cells were incubated with different concentrations of BEPS-IA, and then the cells were stained with methyl green, followed by counting under light microscopy (magnification, ×40). As shown in Figure 7 and Figure S3, treatment with BEPS-IA increased the apoptotic staining in a dose-dependent manner (4.1% ± 1.3% in the control, 12% ± 2.5% in the 50 μg/mL group, 16% ± 5.2% in the 100 μg/mL group, 23% ± 2.3% in the 200 μg/mL group). These data corroborate the BEPS-IA-induced sub-G1 apoptotic fractions determined with flow cytometry shown above. These data corroborate the BEPS-IA-induced sub-G1 apoptotic fractions determined with flow cytometry shown above.

### 2.8. Effect of BEPS-IA on Protein Expression of p53, p21, Bcl-2 and Bax in HepG2 Cells

**Figure 8:**
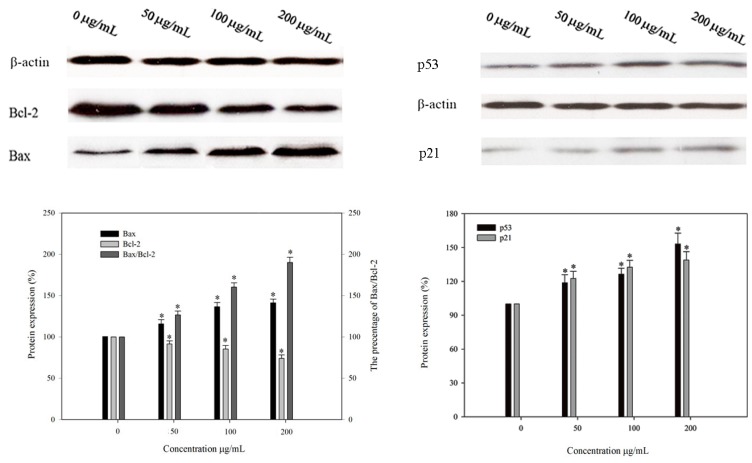
Effects of *B. exarata* polysaccharide-protein complex BEPS-IA on the expression of p53, p21, Bcl-2 and Bax proteins associated with cell cycle and apoptosis in HepG2 cells. Western blot analysis was performed in triplicate per experimental point, and the relative expression of protein was quantified densitometrically (%); β-actin was used as reference control. Values marked with * are significantly different compared to the control (*p* < 0.05).

To gain further information on the mechanism of anti-proliferation induced by BEPS-IA, the expression of p53, p21, Bcl-2, Bax were examined in HepG2 cells. The levels of β-Actin served as an internal control. By studying the results of western blot, we observed that the expression of Bcl-2 was down-regulated, but the level of p53, p21 and Bax were up-regulated, compared to the control (Figure 8). Therefore, we postulated that the possibly mechanism of BEPS-IA-induced anti-proliferation in HepG2 cells by the increase of p53, p21 and the low ratio of Bcl-2 to Bax modulated.

*Bullacta exarata* has been consumed in Asia, not only as a part of the normal diet, but also as a traditional Chinese medicine with liver- and kidney-benefitting functions [3,4,5,6]. Human hepatocellular carcinomas is known to be the most common malignancy and the third leading cause of cancer mortality in people in both developed and developing countries [19]. Polysaccharide-protein complex is one of the major active compounds of *B. exarata*, the antitumor activity of polysaccharide-protein complex has never been reported before. Our study was the first to show that a polysaccharide-protein complex BEPS-IA from *B. exarata* inhibited the growth of human hepatocellular carcinoma HepG2 cells. The results demonstrated that BEPS-IA could induce a significant dose-dependent inhibition of HepG2 cell growth (Figure 2). The IC_50_ of BEPS-IA was 112.4 μg/mL. Further analysis revealed BEPS-IA inhibited the growth of HepG2 cells mainly through blocking cell cycle progression at G1 phase as well as via inducing cell apoptosis.

Furthermore, we explored the molecular mechanisms underlying the BEPS-IA induced cell cycle arresting in HepG2 cells. Previous studies showed that chemo preventive agents were capable of inducing cell cycle blocking and apoptosis [20]. It is also widely recognized that p53 serves as a key player in mediating cell response to various stresses, exerting its function mainly through inducing or suppressing a number of transcriptional regulatory proteins involved in cell cycle arrest and apoptosis, such as the cyclin-dependent kinase (CDKs) inhibitor p21, for the cell cycle arrest and the proapoptotic protein Bax for inducing apoptosis [21]. P21 is a very important checkpoint transcriptional regulatory protein in the cell cycle, which is also regulated by the transcription of p53; it can repair damaged cells by stopping DNA synthesis and inactivate the nuclear antigen in proliferating cells [22]. Our data showed that BEPS-IA up-regulated p53 and p21 expression in a dose-dependent manner (Figure 4). These data suggested that the cell cycle arrest induced by BEPS-IA might be mediated through the regulation of the cell cycle regulating factors, in particular p53.

Alternatively, p53 can also induce apoptosis as a mediator through expression of Bcl-2 and Bax [23]. It has been shown previously that Bcl-2 family proteins are important regulators of the apoptosis [24]. Bax associates with Bcl-2 protein and down-regulation of Bcl-2 favors the formation of Bax homodimer that increases the rate of apoptosis and sensitivity of cells to apoptotic stimuli [25]. The Bax/Bcl-2 ratio was found to be elevated at the time when most prominent apoptosis occurred. Our data demonstrated that BEPS-IA treatment resulted in a significant decrease of Bcl-2 expression and an increase of Bax, thus the calculated Bax/Bcl-2 ratio was markedly higher in the BEPS-IA -treatment group compared to the control group. Increasing evidence demonstrated that the Bax/Bcl-2 ratio determines the susceptibility of cells to the induced apoptosis [24,25]. Therefore, we concur that the growth inhibition process occurring in in vitro cultured HepG2 cell lines after BEPS-IA treatment may be associated with the up-regulation of p53, p21 and bax expression and down-regulation of bcl-2 expression, suggesting that p53 was involved in cell cycle arrest and apoptosis regulation of BEPS-IA.

The structure of the polysaccharide is strongly related to its antitumor activity. Previous research has mainly focused on the structure of main chain structure, branching degree, helical conformation, molecular weight, and the monosaccharide composition. Generally, polysaccharides of molecular weights between 400 and 800 kDa appeared to be more effective than those of low molecular weight in suppressing tumor cell growth [26]. In addition, the amount of protein groups has been reported to play role for antitumor activity of polysaccharide-protein complexes [12]. Moreover, according to the report, polysaccharides with β-glucan such as (1→3)-linked-β-d-glucose backbone chain exerted a relatively higher antiproliferation activity [27]. However, in practice, it is difficult to make a correlation between antitumor activity of purified polysaccharide-protein complex and their structures. There are some other antitumor polysaccharides with different chemical structures, such as α-glucan or low molecular weights [28]. Thus, the antitumor activity of tested polysaccharide-protein complex is not a function of a single factor but a combination a many factors such as amount of protein groups, monosaccharide residues ratio, and type of sugar residues. To our knowledge, there are few reports of polysaccharides with a structure similar to BEPS-IA displaying an antitumor effect and having apoptosis-inducing activity. Our research will facilitate the understanding of the structural basis of the polysaccharides with antitumor effects and their antitumor mechanisms.

## 3. Materials and Methods

### 3.1. Materials and Reagents

*Bullacta exarata* snails were obtained from Rulong Seafood Co., Ltd. (Zhejiang, China). Monosaccharide standards (arabinose, rhamnose, fucose, xylose, galactose, glucose and mannose), disaccharide lactose, sodium dodecyl sulfate (SDS), ethylendiamine tetraacetic acid (EDTA), bovine serum albumin (BSA), papain, cysteine (Cys) and nonfat milk powder were obtained from Sigma Chemical Company (St. Louis, MO, USA). MTS was from Promega (Madison, WI, USA). Anti-Bax antibody (rabbit polyclonal) was purchased from Santa Cruz Biotechnology (Santa Cruz, CA, USA). Anti-P53 antibody (mouse monoclonal) and anti-Bcl-2 antibody (mouse monoclonal) were purchased from Abcam (Cambridge, MA, USA). HRP-linked anti-mouse IgG secondary antibody was purchased from Cell Signaling Technologies (Danvers, MA, USA). All other chemicals used were analytical grade.

### 3.2. Polysaccharide-Protein Complex BEPS-IA Extraction

The BEPS-IA was isolated and purified by our previously described procedure [2]. Fresh or deep-frozen *Bullacta exarata* (500 g) was defatted by extraction for 24 h using for acetone (3L). Samples of defatted, dried, and powdered *Bullacta exarata* were treated with papain (1200 U·g^−1^) for 24 h at 200 rpm at 60 °C. The extracts were combined, centrifugated, dialyzed, concentrated, and polysaccharides were precipitated with four volumes of 96% ethanol. The precipitates were washed with 96% ethanol and acetone and air-dried. The crude polysaccharide preparation was applied onto a DEAE column (2.5 cm × 30 cm, Bio-Rad, Hercules, CA, USA), followed with a Sephacryl S-300 gel filtration column (1.6 cm × 100 cm). The final two fractions, named as BEPS-IA and BEPS-IB, were desalted and lyophilized as previously reported [2]. In this study, the polysaccharide conjugate BEPS-IA, with an average molecular weight of about 127 kDa, was collected for further study.

### 3.3. Analytical Procedures

Total carbohydrates were quantified using the phenol-sulfuric acid method [29]. The monosaccharide composition was analyzed by the PMP-HPLC method. 13 Sulfate group determination was carried out by the BaCl2/gelatin method and ion-exchange chromatography [30]. The contentration of proteins was quantified by the method of Lowry and the amino acid constituents were analyzed by the HPLC AccQ method [31]. Tryptophan can be obtained by alkaline hydrolysis and UV detection at 280 nm [32]. BEPS-IA was imaged by AFM method according to previously described procedure [33]. Topographic observations were performed using the Nano Scope III atomic force microscope (Digital Instruments, Santa Barbara, CA, USA) and collected by tapping mode atomic force microscopy (Tm AFM).

### 3.4. Methylation Analyses

Methylation of BEPS-IA was carried out three times using the method described by Souza et al. [34]. The methylated polysaccharides were confirmed by FTIR spectroscopy. The complete methylated products were hydrolyzed, reduced and acetylated as described earlier. The resulting alditol acetates were subjected to GC and GC-MS analysis. Qualitative and quantitative analyses were performed on gas chromatography–mass spectrometry (GC-MS, Finnigan Trace, Thermo Electron Finnigan Co. Ltd., Waltham, MA, USA) with helium as carrier gas (2 mL/min). The GC column was an OV1701 (30 m, internal diameter 0.25 mm, film thickness 0.25 μm) at a temperature program from 140 to 280 °C with a rate of 3 °C/min. The rate of helium carrier gas was at a rate of 3.0 mL/min. The quantification for molar ratio for each *O*-methylated alditol acetate was calibrated using the peak area and response factor of the FID in GC.

### 3.5. FI-IR and NMR Analyses

Nuclear magnetic resonance (NMR) spectra (^1^H, ^13^C) for solutions of substances in D_2_O were obtained on a 500 MHz Avance™ 500 spectrometer (Bruker, Berlin, Germany) at 60 °C [35]. FTIR spectra (KBr disc) were recorded with a Nicolet 5700 FT-IR (Thermo Electron Corporation, Madison, WI, USA) spectrophotometer for detecting functional groups [36].

### 3.6. Cell Culture

The human hepatocellular carcinoma HepG2 cell line (ATCC, Rockville, MD, USA) was grown in monolayer in minimum essential medium alpha medium (MEM) containing 50 units/mL penicillin, 50 μg/mL streptomycin, 100 μg/mL gentamicin, 10 mM Hepes, 5 μg/mL insulin, 2 μg/mL glucagon, 0.05 μg/mL hydrocortisone and 5% fetal bovine serum (Gibco, Life Technologies, Grand Island, NY, USA) in a humidified atmosphere containing 5% CO_2_ at 37 °C [37].

### 3.7. Cytotoxicity Measurements

To estimate cell cytotoxicity, cells (HepG2) were seeded (4.0 × 10^4^/well) in 96-well flat-bottom plate at 37 °C in a 5% CO_2_ incubator. After 24 h, the medium was removed and replaced by a fresh medium containing the different concentrations of the polysaccharide BEPS-IA for additional 24 h at 37 °C in a 5% CO_2_ incubator. Blank wells contained 100 μL of growth medium with no cells and control cultures (CK) received the extraction solution minus the extracts. Cytotoxicity was determined by the methylene blue assay and measured by a 10% reduction of absorbance at 570 nm reading for each concentration compared to the control [38,39].

### 3.8. Measurement of Cell Proliferation

To estimate cell proliferation, cells (HepG2) were seeded (2.5 × 10^4^/well) in 96-well flat-bottom plate at 37 °C in a 5% CO_2_ incubator. After 4 h, the medium was removed and replaced by a fresh medium containing the different concentrations of the polysaccharide BEPS-IA for additional 72 h at 37 °C in a 5% CO_2_ incubator. Blank wells contained 100 μL of growth medium with no cells and control cultures (CK) received the extraction solution minus the extracts. The common antitumor drug 5-fluorouracil (5-FU) was used as a positive control. Cell proliferation was determined by the methylene blue assay and measured by the absorbance at the 570 nm reading for each concentration compared to the control [39].

### 3.9. TUNEL Assay

In vitro induction of apoptosis by BEPS-IA treatment was determined by the TUNEL assay, with an Apoptosis Detection Kit (Biovision Research Products, Mountain View, CA, USA). HepG2 cells were harvested and cytospun on slides with concentration of 1 × 10^5^ cells/mL. TUNEL staining was performed using the detection kit according to the manufacturer’s instructions. The apoptotic cells were counted with a light microscope (Olympus, C5060-ADU, Tokyo, Japan) and the results are reported as the mean of the three experiments.

### 3.10. Cell Cycle Analysis

Cells were cultured with BEPS-IA (50, 100, 200 μg/mL) for 24 h, while the control group received only growth medium. The cells were harvested and washed in ice-cold PBS for three times. Cells were then fixed with ice-cold 70% methanol, and DNA was stained with propidium iodide (50 mg/mL). Cell cycle analysis was performed with flow cytometer (BD FACSVerse™, BD Biosciences, Franklin Lakes, NJ, USA). ModFitL T V2.0 software was used to determine the cell cycle phase distribution after debris exclusion. The sub-G1/G0 cell fraction was considered representative of apoptotic cells.

### 3.11. Western Blotting Analysis

BEPS-IA treated cells were lysed in solubilization buffer (50 mM Tris, pH 7.4; 1% Igepal; 150 mM sodium chloride; 1 mM EDTA) with protease inhibitors (1 μg/mL aprotinin; 1 μg/mL leupeptin; 1 μg/mL pepstain; 1 mM phenylmethylsulfonyl fluoride (PMSF); 1 mM sodium orthovanadate; 1 mM sodium fluoride) for 30 min at 4 °C, and insoluble materials was removed by centrifugation at 12,000× *g* for 10 min at 4 °C. The supernatant was collected and the protein concentration was determined by Sigma Diagnostics Micro Protein Determination Kit and Dynex Microplate Reader (Dynex Technologies, Chantilly, VA, USA). Equal amounts of protein (60 mg) were loaded in each lane and separated by 12% SDS-PAGE. The proteins were transferred to Immobilon-p transfer membranes. The membranes were blocked with 3% non-fat dry milk in TBS containing 0.1% Tween 20, and then incubated at room temperature for 1 h in the presence of each antibody (Anti-Bax antibody, Anti-P53 antibody and anti-Bcl-2 antibody). The membrane was washed three times with 0.1% Tween 20 in PBS and stained with HRP-linked anti-mouse IgG secondary antibody. β-Actin was used as internal control. The ECL system (Amersham Biosciences, Buckinghamshire, UK) was used for detection [40].

### 3.12. Statistical Analyses

Experiments were performed at least three times with similar results. All results were presented as mean ± SD. Statistical differences were evaluated using SPSS software (SPSS Inc., Chicago, IL, USA) and considered to be significant when *p* < 0.05.

## 4. Conclusions

In summary, a novel protein-bound polysaccharide BEPS-IA was extracted and purified from *Bullacta exarata*. The glycoprotein was mainly composed of mannose and Glc in a molar ratio of about 1:2, with an average molecular weight of about 127 kDa. The glycosyl residues of BEPS-IA mainly linked by α-configuration glycosidic bonds, and theirs repeating unit of structure were proposed as described above. Antitumor activity tests conducted in vitro revealed that BEPS-IA exhibited an effective HepG2 breast cancer cell growth inhibition. The inhibitory action, does-dependent manner, was mainly caused by G1 cell cycle arrest and apoptosis. And the mechanism was associated with up-regulation of p53, p21 and Bax, down-regulation of Bcl-2. Therefore, the present studies demonstrated that BEPS-IA would be a new adjuvant chemotherapeutic and chemo-preventive strategy against human hepatocellular carcinoma HepG2 cells in vitro. However, due to only one cell line in our present study, further investigations on more cell lines supporting the findings are currently in progress.

## Figures and Tables

**Table 1:** The amino acid composition of BEPS-IA.

Amino Acid	Concentration (mg/g) ^a^
Asparagine	16.73 ± 1.37
Serine	5.33 ± 2.11
Glutamic acid	12.27 ± 3.79
Glycine	22.14 ± 0.68
Arginine	4.12 ± 1.61
Threonine ^b^	1.04 ± 2.14
Alanine	9.26 ± 2.12
Proline	5.12 ± 2.01
Cysteine	4.28 ± 0.17
Valine ^b^	8.95 ± 1.42
Methionine ^b^	1.21 ± 2.04
Lysine ^b^	2.41 ± 0.26
Leucine ^b^	6.32 ± 0.24
Total amino acids	99.18 ± 6.43
Proportion of the essential amino acids (%)	20.09 ± 5.62

^a^ Data are shown as mean ± SD (*n* = 3); ^b^ Essential amino acids.

**Table 2:** GC-MS of alditol acetate derivatives from the methylated product of BEPS-IA.

Methylated Sugar	Molar Ratio	Mass Fragment (*m*/*z*) ^b^	Type of Linkage
2,3,6-Tri-*O*-Me-Glc ^a^	6.46	45, 87, 101, 113, 117, 161, 233	→4)-Glc*p*-(1→
2,4,-Di-*O*-Me-Man	1.13	71, 87, 99, 101, 189	→3,6)-Man*p*-(1→
2,3,4,-Tri-*O*-Me-Man	0.87	45, 71, 87, 101, 129, 161, 189	→6)-Man*p*-(1→
2,3,4,6-Tetra-*O*-Me-Man	0.91	28, 43, 71, 87, 101, 117, 129, 145, 161,205	Man*p*-(1→

^a^ 2,3,6-Tri-*O*-Me-Glc = 1,5,4-tri-*O*-acetyl-2,3,6-tri-*O*-methyl-d-glucitol. ^b^ Equipped with a OV1701 capillary column (30 m × 0.25 mm internal diameter) using a temperature program from 150 °C (2 min) to 250 °C (5 min) at 3 °C·min^−1^.

## Supplementary Materials

Supplementary materials are available online.

## Abbreviations

BEPS-IA	purified *Bullacta exarata* polysaccharides of molecular weight 127 kDa
AFM	Atomic Force Microscopy
NMR	Nuclear Magnetic Resonance
PMP	1-phenyl-3-methyl-5-pyrazolone
CK	Control culture
IC_50_	inhibitory concentration representing the concentration at which cell viability was reduced by 50%
HepG2	human hepatocellular carcinoma cell
MEM	Medium Alpha Medium
Gal	galactose
Man	mannose
Glc	glucose
GlcUA	glucuronic acid
Arb	arabinose
Rha	rhamnose
Xyl	xylose
GalN	galactosamine
GlcN	glucosamine
Fuc	fucose
