# Supplementary material for: A Novel Polysaccharide Conjugate from Bullacta exarata Induces G1-Phase Arrest and Apoptosis in Human Hepatocellular Carcinoma HepG2 Cells
Source: Molecules. 2017 Mar 1;22(3):384. doi: 10.3390/molecules22030384 (PMC6155380; doi:10.3390/molecules22030384)
Supplement: Supplementary file 1 [file molecules-22-00384-s001.pdf]

A novel polysaccharide conjugate from *Bullacta exarata* induces G1-phase arrest and apoptosis in human hepatocellular carcinoma HepG2 cells

Ningbo Liao [1], Liang Sun [1], Jiang Chen [1†],Jianjun Zhong [2], Yanjun Zhang [1] and Ronghua Zhang [1††]

[1] Department of Nutrition and Food Safety, Zhejiang Provincial Center for Disease Control and Prevention, Hangzhou 310006, China

[2] College of Biosystem Engineering and Food Science, Zhejiang University, Hangzhou 310058, China;

Supporting information

Table S1. Chemical compositions and contents of carbohydrate, protein, M_W and sulfonic acid in BEPS-IA

Item	Content [a]
Yield (%) [b]	1.03 ± 0.24
Protein (%)	2.72 ± 0.89
Neutral sugar (%)	76.29 ± 6.31
Sulfonic acid (%)	0.47 ± 0.07
M_W(kDa) [c]	~127

[a] Results are presented as mean ± SD (n=3).

[b] Data are expressed in g/100g dry weight

[c] Molecular weight

Figure S1. Cytotoxicity against HepG2 cells by *B. exarata* polysaccharide-protein complex BEPS-IA (mean ± SD, n = 3).

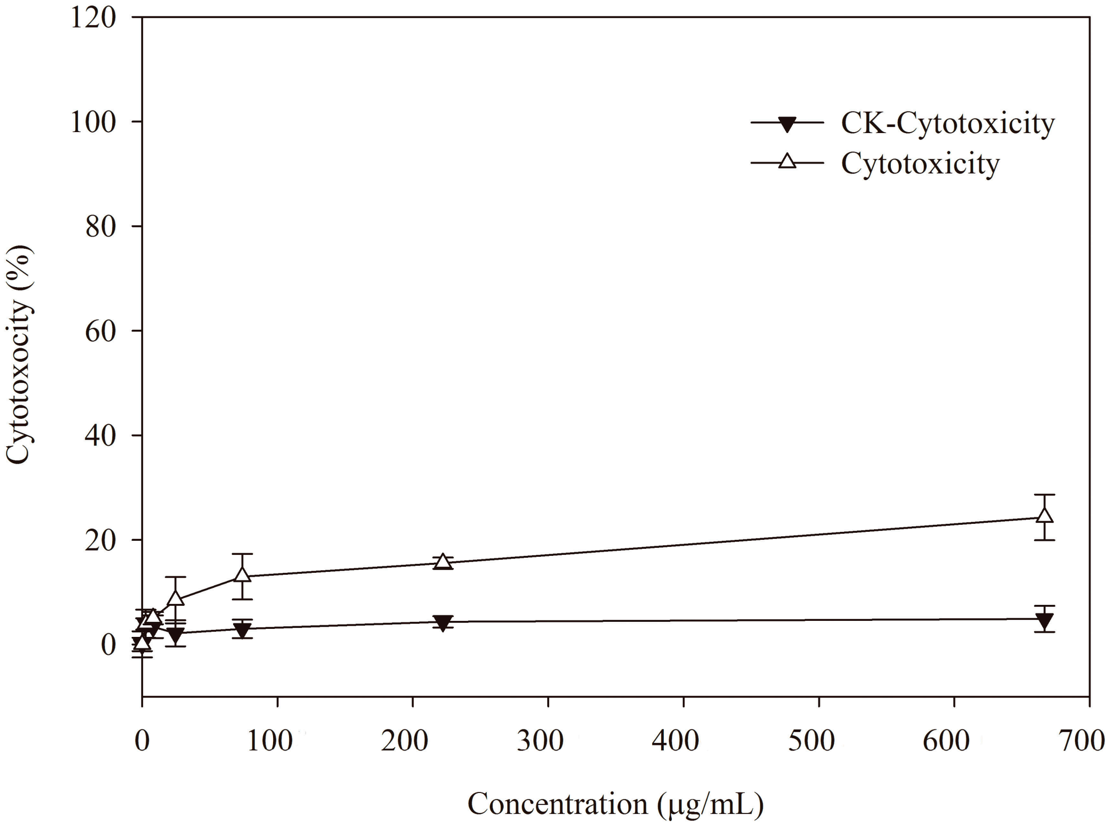

CK, Control of cytotoxicity. Values marked with * are significantly different compared to the control ($p < 0.05$).

Figure S2. Effect of the *B.exarata* protein-bound polysaccharide BEPS-IA on HepG2 cell cycle progression.

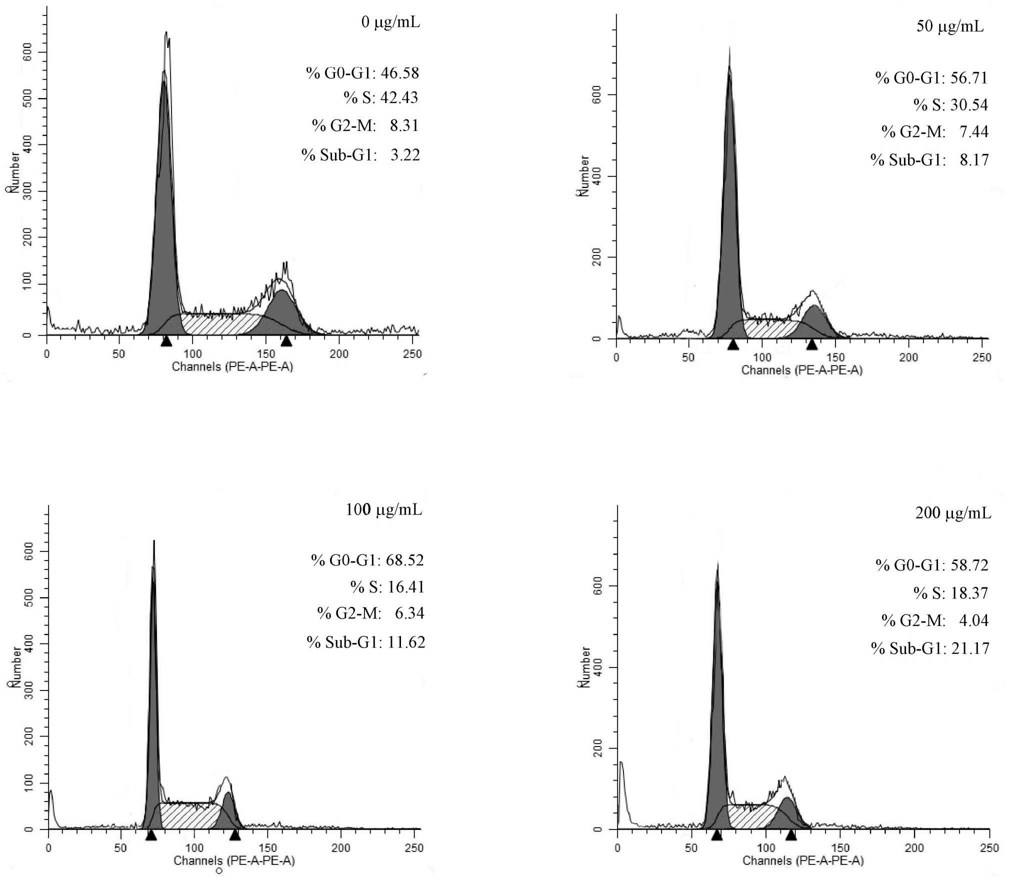

Cells were cultured for 24 h with BEPS-IA (0, 50, 100 and 200 µg/mL, respectively). Untreated cells (0 µg/mL) were used as control. Cell cycle was analyzed with flow cytometry. One representative of three repeat experiments was shown here.

Figure S3. TUNEL staining of HepG2 cells.

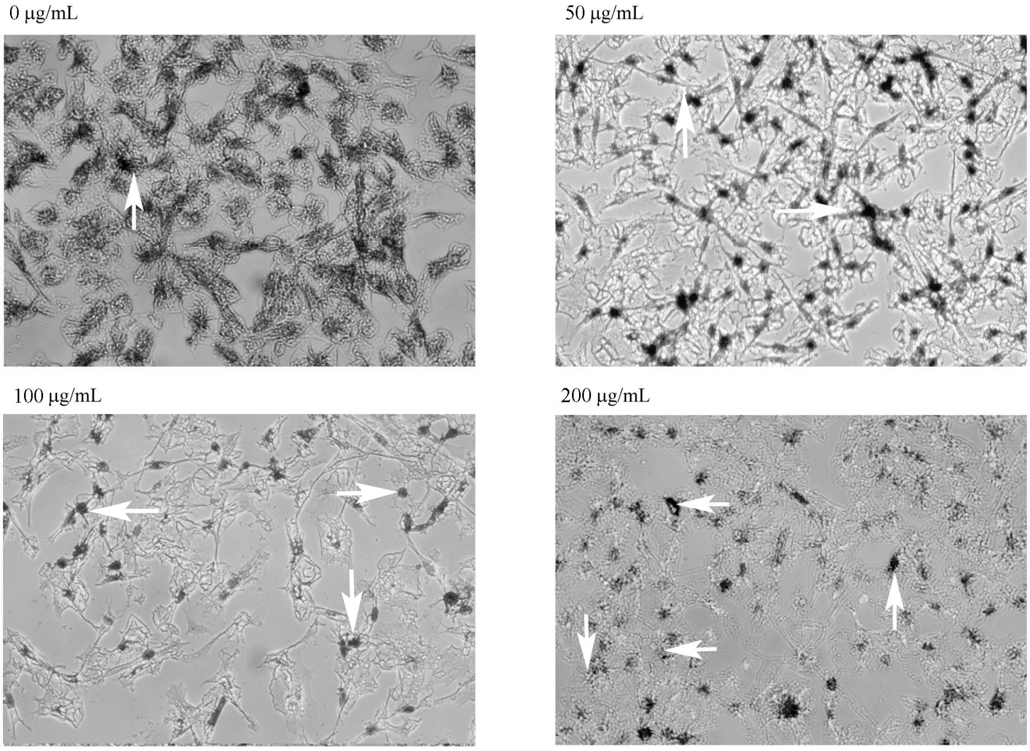

Cells were cultured for 24 h with polysaccharide: 50, 100 and 200 μg/mL. Untreated cells (0 μg/mL) were used as control. After treatment, cells were isolated and prepared for measurement of TUNEL staining using light microscopy (magnification, × 40). Arrows indicate apoptotic cells. Figures are representative of three separate experiments.
